# Follow-up of 2003 Human West Nile Virus Infections, Denver, Colorado

**DOI:** 10.3201/eid1207.051399

**Published:** 2006-07

**Authors:** Jennifer L. Patnaik, Heath Harmon, Richard L. Vogt

**Affiliations:** * Tri-County Health Department, Greenwood Village, Colorado, USA;; † Boulder County Public Health, Boulder, Colorado, USA

**Keywords:** West Nile virus, epidemic, meningitis, encephalitis, fever

## Abstract

Tri-County Health Department and Boulder County Public Health conducted a follow-up study of all nonfatal West Nile virus (WNV) cases reported during 2003 in 4 metropolitan Denver, Colorado, counties. Self-reported patient information was obtained ≈6 months after onset. A total of 656 (81.2%) eligible WNV patients are included in this study.

In 2003, Colorado experienced a large West Nile virus (WNV) epidemic, which accounted for 29.9% of the nation's 9,862 reported WNV infections (*1*). Tri-County Health Department, which serves Adams, Arapahoe, and Douglas counties, and Boulder County Public Health collaborated to conduct a follow-up study of all WNV cases reported in these 4 counties in 2003. We conducted this follow-up study with 3 objectives: 1) to identify potential risk factors for developing neuroinvasive disease, 2) to describe the symptoms of patients 6 months after onset, and 3) to describe healthcare utilization and impact on daily activities associated with all types of WNV infection.

## The Study

Since 2002, healthcare providers and laboratories have been required to report patients with laboratory evidence of acute WNV infection in Colorado. Patients were included in this study if WNV-specific immunoglobulin M (IgM) antibodies were found in either cerebrospinal fluid (CSF) or serum by enzyme-linked immunosorbent assay, or symptoms later developed in blood donors with a positive nucleic acid test result. In addition to laboratory confirmation, patients had to meet one of the following case definitions to be included in the study: 1) encephalitis cases required a physician's diagnosis and clinical manifestation of encephalitis, including mental status changes, delirium, disorientation, or coma; 2) meningitis cases required a physician's diagnosis or clinical exhibition of meningitis and abnormal CSF findings consistent with viral meningitis; and 3) fever cases required mild to moderate illness without clinical or laboratory evidence of central nervous system involvement. A compatible illness of WNV fever was defined as symptoms consisting of >2 of the following occurring within 90 days of testing: fever, headache, chills, myalgia, arthralgia, rash, lymphadenopathy, muscle weakness, or severe malaise. Any patient with a positive IgM test result on CSF was considered to have neuroinvasive disease.

Self-reported patient information was solicited through a standardized survey sent to 808 patients with nonfatal cases. Cases of meningitis and encephalitis were compared with cases of WNV fever. Measures of association between diagnosis and relevant patient characteristics were determined by Wald χ^2^, odds ratios, and associated 95% confidence intervals for categorical variables and analysis of variance (ANOVA) testing for continuous variables. Multivariate logistic regression modeling was used to test for potential predictors of more severe disease at time of diagnosis. Variables were considered significant at the p = 0.05 level. Data were entered into EpiInfo 2002 (available from http://www.cdc.gov/epiinfo/) and analyzed with SAS version 9.1 software (SAS Institute, Inc., Cary, NC, USA).

A total of 656 (81.2%) patients completed the survey: 52.1% were female, 42.8% were >50 years of age, 80.9% had a diagnosis of fever, 12.8% had a diagnosis of meningitis, and 6.3% had a diagnosis of encephalitis. Nineteen cases were detected through blood donor screening, and all were categorized as uncomplicated fever cases. Nonrespondents were less likely to be female (42.1%, p = 0.0259) and >50 years of age (27.6%, p = 0.0007) but were similar by diagnosis category (p = 0.5846).

Mean ages by diagnosis were 60 years for encephalitis patients, 48 years for meningitis patients, and 46 years for fever patients. Encephalitis patients were significantly older than meningitis and fever patients (p<0.0001). The median period between onset of illness and completion of the follow-up survey was 178 days (range 102–299 days); 80% responded within 5–7 months after illness onset.

The overall prevalences of several chronic conditions and treatments are shown in Table 1. After adjustment for sex and age >50 years, encephalitis patients were significantly more likely than fever patients to report having several chronic conditions and to report having been on chemotherapy. Meningitis patients were more likely than fever patients to report having cancer and to have undergone chemotherapy.

**Table 1:** Prevalence of chronic health conditions and medication use among West Nile virus (WNV) study patients and multivariate model of predictors of more severe illness*

Condition	All WNV patients (n = 656); prevalence, %	Meningitis patients (n = 84); adjusted OR (95% CI)†	Encephalitis patients (n = 41); adjusted OR (95% CI)†
High blood pressure	12.2	1.0 (0.4–2.1)	2.1 (1.0–4.6)
Diabetes	6.1	0.8 (0.3–2.5)	2.6 (1.0–6.5)
Heart disease	3.8	1.4 (0.5–4.5)	2.7 (0.9–8.2)
Cancer	1.5	6.6 (1.6–27.5)	7.5 (1.2–45.4)
Kidney disease	1.2	2.3 (0.2–22.9)	24.9 (4.7–132.5)
Steroids	3.0	1.3 (0.4–4.6)	1.8 (0.4–8.5)
Chemotherapy	1.4	7.7 (1.5–40.0)	25.9 (4.2–159.7)

*CI, confidence interval; OR, odds ratio. †Adjusted for sex and age >50 y.

Symptom duration was reported as >3 months for 48.7% of encephalitis patients, 26.2% of meningitis patients, and 20.3% of fever patients (Table 2). Muscle weakness and muscular pain at time of follow-up were reported by more than one third of encephalitis patients (Table 3). No notable differences in symptoms were reported based on the difference in the interval between onset date and date of completing the follow-up survey.

**Table 2:** Duration of symptoms for West Nile virus study patients

Duration of symptoms, d	Fever patients (n = 531); no. (%)	Meningitis patients (n = 84); no. (%)	Encephalitis patients (n = 41), no. (%)
<30	241 (46.3)	20 (23.8)	8 (20.5)
31–90	174 (33.4)	42 (50.0)	12 (30.8)
>90	106 (20.3)	22 (26.2)*	19 (48.7)*

*Significantly different than among fever patients; p<0.05 (applies to overall distribution of 3 categories).

**Table 3:** Symptoms ever experienced and still experiencing at time of follow-up for West Nile virus study patients

Symptom	Fever patients (n = 531)		Meningitis patients (n = 84)		Encephalitis patients (n = 41)	
	Ever, %	At follow-up, %	Ever, %	At follow-up, %	Ever, %	At follow-up, %
Muscle weakness	80.4	12.2	96.3	28.0*	92.7	46.3*
Muscle pain	85.9	12.1	92.6	14.8	76.3	39.5*
Headache	88.6	11.8	91.5	19.5	65.0*	12.5
Stiff neck	78.6	10.4	84.1	12.2	59.0*	12.8
Sensitivity to light	52.6	5.6	71.6*	11.1	63.4	12.2

*Significantly different than among fever patients; p<0.05.

Hospital admission was significantly more common among encephalitis (97.6%) and meningitis (91.7%) patients than fever patients (13.9%). The mean length of stay for all hospitalized patients was 11 days (range 1–165 days) and was significantly higher for encephalitis patients (20 days) than meningitis patients (10 days) and fever patients (7 days). Significantly more encephalitis and meningitis patients sought physical therapy (65.9% and 34.9%, respectively), occupational therapy (50.0% and 18.3%, respectively), and speech therapy (30.8% and 10.8%, respectively) than fever patients. Among fever patients, 6.6% reported receiving at least 1 of the 3 therapies.

Missing time from work was reported by most all categories of cases. For the 485 patients who were working at the time of illness onset, encephalitis patients and meningitis patients were significantly more likely to report missing work (100.0% and 98.3%, respectively) than fever patients (78.9%). The median number of work days missed was significantly higher among encephalitis patients (65 days) and meningitis patients (51 days) than fever patients (16 days). In addition, 91.0% of all patients reported that their routine daily activities were prevented by their WNV infection.

## Conclusions

This study characterizes the severe impact that WNV infection had on all age groups and categories of WNV illness in a defined population-based cohort of 656 nonfatal infections. Our study results corroborated findings from previous studies that older age is predictive of more severe WNV illness, such as encephalitis (*2**–**4*) and death (*2**,**4**–**7*). In our study, the mean age of meningitis patients did not differ significantly from that of fever patients.

Additionally, we identified several preexisting medical conditions, as well as prior utilization of chemotherapy, that may predispose infected persons to the development of encephalitis or meningitis. The risk for encephalitis has been found to be higher among organ transplant recipients (*8*); however, the literature is inconsistent regarding whether preexisting medical conditions are predictive of neuroinvasive disease (*2**,**4**,**7**,**9*). The studies that did not detect such associations used different comparison groups than did our study and were limited by small sample size or low prevalence of these chronic medical conditions.

Only 1 other study has characterized the clinical spectrum of symptom duration among West Nile fever patients and missed work or school days (*10*). This study of 98 fever patients found that 39% had ongoing symptoms after an average of almost 6 months of follow-up, 82% reported limitations in household activities, and a median number of 10 missed work or school days (*10*). Our fever patients reported a higher number of missed work or school days with a median of 16. Additional studies with objective measures could better elucidate the long-lasting effects of WNV infection.

Because of the nature of self-reported data, both recall bias and misclassification of self-reported information are potential limitations of this study. However, we validated self-reporting of definitive fields such as sex and hospitalization because they were highly correlated with the initial data maintained in our statewide surveillance database.

Another limitation of our study was that a clinical diagnosis of flaccid paralysis or lack thereof was not confirmed in study cases. Estimated rates of flaccid paralysis are low (*2**,**11*) and therefore should not have had a large impact on our study findings. In addition, patients who had died were excluded from the study; therefore, we were not able to characterize this group for preexisting chronic conditions. Our study was limited to reported case-patients who sought medical attention and laboratory testing; therefore, our findings likely represent the more severe spectrum of infections.

Our study demonstrates that WNV infection caused considerable, long-lasting, severe illness during the 2003 Colorado epidemic and that the economic impact in terms of associated healthcare utilization and days of missed work was substantial. Public health officials should intensify prevention messages to help limit the severe manifestations of WNV infection and especially target those at greatest risk for severe disease.
